# Exploring Resilience in Mothers of Adolescents With Intellectual Disabilities in Thailand: A Qualitative Study

**DOI:** 10.1111/jar.70271

**Published:** 2026-06-23

**Authors:** Wattana Tejakum, Maria Truesdale, Deborah Cairns

**Affiliations:** ^1^ School of Health and Wellbeing, College of Medical, Veterinary & Life Science University of Glasgow Glasgow UK; ^2^ Boromarajonani College of Nursing, Bangkok Faculty of Nursing, Praboromarajchanok Institute Bangkok Thailand

**Keywords:** adolescents, intellectual disabilities, mothers, resilience, Thailand

## Abstract

**Background:**

Raising adolescents with intellectual disabilities in Thailand is challenging. Whilst some mothers show resilience, limited understanding exists of the factors within these cultural contexts that contribute to resilience. This qualitative study explores experiences of mothers caring for adolescent children with intellectual disabilities in Thailand.

**Methods:**

Semi‐structured interviews were conducted with 12 Thai mothers of adolescents with intellectual disabilities at the Rajanukul Institute in Thailand. Interview transcripts were analysed using thematic analysis, guided by Braun and Clarke (2006).

**Results:**

Six themes were developed, detailing mothers' resilience journeys: becoming a mother of a child with intellectual disabilities, finding my way, navigating challenges, support systems, holding on to faith, and forward‐thinking strategies.

**Conclusion:**

This study reveals that resilience amongst mothers is a dynamic process shaped by personal attributes, coping strategies, social support, and cultural and spiritual beliefs. These findings highlight the need for culturally sensitive interventions to strengthen parental resilience.

## Lay Summary

- This qualitative study explored the personal experiences of 12 mothers in Thailand raising adolescents with intellectual disabilities, offering deep insights into their resilience.
- The study underscores the importance of culturally specific support, showing how Thai cultural elements, such as the concept of “Thum‐jai” (making peace in your heart), play a role in how these mothers cope.
- Whilst this study provides rich, in‐depth accounts of mothers' experiences during adolescence, it also focuses on a specific stage. Further research could explore how resilience evolves across the lifespan of caregiving for individuals with intellectual disabilities in this cultural context.

## 1 Introduction

Mothers are widely recognised as the primary caregivers for children with intellectual disabilities, a role that often comes with significant emotional, physical, and financial demands (Lee 2013; Gordon and Bila 2023). This role intensifies during the child's transition to adolescence, a critical developmental period that introduces new complexities related to reaching expected milestones, behaviour, sexuality, future planning (Maxey and Beckert 2017; Patton et al. 2018; Goli et al. 2020; Kerr et al. 2023) and a period that may also impact on the mothers' health and wellbeing (Rydzewska et al. 2021). Whilst a considerable body of research has explored the stressors and burdens associated with caring for a child with intellectual disabilities, there is growing recognition of the remarkable resilience demonstrated by many parents (Peer and Hillman 2014; Beighton and Wills 2019; Alsharaydeh et al. 2023; Yang 2025).

However, resilience is a complex and evolving construct. Early conceptualisations, Rutter (1987), framed resilience as an individual capacity to achieve positive outcomes despite significant risk exposure. This perspective positioned resilience as a relatively stable characteristic that enabled individuals to “withstand” adversity. Over time, rather than viewing resilience as a stable personality trait, contemporary scholarship conceptualises it as a dynamic and contextually embedded process of adaptation in the face of adversity (McCubbin et al. 1996). Similarly, Luthar and Cicchetti (2000) reframed resilience as a dynamic process of positive adaptation within the context of significant adversity, emphasising developmental trajectories and the interaction between risk and protective factors. More recent scholarship has further expanded the concept beyond the individual. Masten (2018) conceptualised resilience as the adaptive capacity of systems, individuals, families, and communities to function effectively under stress, highlighting its ecological and relational dimensions. Walsh (2015) similarly advanced a family resilience framework, underscoring shared meaning‐making, relational processes, and collective coping strategies.

In line with this conceptualisation, resilience refers to the capacity to navigate significant adversity, maintain positive functioning, and even experience growth in the face of chronic stress (Herrman et al. 2011; Ungar 2018). Understanding the factors that foster resilience is crucial for developing effective support systems and interventions to enhance the well‐being of these mothers.

Whilst caregiving challenges are universal, the pathways to resilience are profoundly shaped by local cultural values, social structures and belief systems (Ungar 2008). In Thailand, where societal norms are deeply interwoven with Buddhist philosophy and strong familial bonds, these cultural elements provide a unique lens for understanding the caregiving experience (Klangrit et al. 2025). In the Thai cultural context, specific societal norms, family structures, and spiritual beliefs may uniquely influence how mothers perceive challenges, access support, and develop coping strategies. Therefore, this study seeks to explore these experiences within their specific cultural milieu.

This qualitative study aimed to explore the experiences of mothers raising adolescents with intellectual disabilities in Thailand, with a particular focus on understanding the factors that contribute to their resilience to their lived experiences. A qualitative approach offered a platform for these mothers to articulate their perspectives, share their stories, and illuminate the complex interplay of the personal, familial, social, and cultural factors that shape their resilience, an area that has not been previously explored in the Thai context.

## 2 Method

### 2.1 Research Design

This study employed a qualitative research design to explore the experiences and resilience of mothers of adolescents with intellectual disabilities in Thailand. Qualitative methodology was chosen for its suitability in gaining an in‐depth understanding of complex human experiences, perspectives, and the meanings that individuals attribute to them (Braun and Clarke 2006; Creswell 2017).

### 2.2 Participants

Thai mothers who have an adolescent (10–18 years old) son/daughter with an intellectual disability living at home. The study population comprised 12 mothers of adolescents with intellectual disabilities who resided in Thailand and 13 sons/daughters with intellectual disabilities. Mothers were recruited via the Rajanukul Institute, Department of Mental Health, Ministry of Public Health, Bangkok. The Rajanukul Institute is a tertiary care centre for child and adolescent psychiatry that provides services to children and adolescents with intellectual disabilities and other developmental, behavioural, or emotional problems. The detailed demographic information is provided in Table S1.

### 2.3 Data Collection

Data were collected through individual, in‐depth semi‐structured interviews with the participants. An interview guide was developed and used to facilitate the interview process, whilst also providing the flexibility to develop themes and allow participants to express themselves in their own words. A historical approach was taken, with the researcher asking questions about their son's or daughter's early years up to the present day. The development of this instrument was a rigorous, multistage process designed to ensure its relevance, cultural sensitivity, and theoretical grounding. An initial set of open‐ended questions was drafted based on the key concepts identified in the literature on caregiver resilience (Luthar and Cicchetti 2000; Black and Lobo 2008) (see Table S2). This guide was explicitly structured to probe the core components of McCubbin's resilience model (McCubbin et al. 1996), which conceptualises family adaptation as the outcome of dynamic interactions between accumulated stressors, available internal and external resources, cognitive appraisal processes, and problem‐solving strategies. This model was subsequently used as a guiding framework, as it offers a comprehensive, empirically supported structure for examining resilience within family caregiving contexts, aligning closely with the study's focus on how mothers of adolescents with intellectual disabilities in Thailand adapt to ongoing caregiving challenges.

Questions were designed to elicit narratives about their overall experiences (see Table S3), including stressors, available resources, the family's perception of their situation, and specific coping strategies. Questions within the interview guide included: “Can you share your experiences of raising an adolescent with an intellectual disability?”, “Can you share any moments when you've felt particularly resilient in your role as a mother of an adolescent with an intellectual disability?”. The draft guide was reviewed by two other researchers specialising in families of people with intellectual disabilities to refine the questions for cultural appropriateness, clarity, and linguistic nuance. Finally, the interview guide was piloted with two mothers who met the study's inclusion criteria. The pilot study confirmed that the questions were clear, understandable, and effective in eliciting rich, narrative responses. No changes to the format are required. To maximise the dataset, two pilot participants were included in the final sample with their consent, bringing the total number of interviews to 12. To ensure that cultural and linguistic subtleties were fully captured, all interviews were conducted in Thai by a native Thai‐speaking researcher.

Ethical approval was obtained from the University of Glasgow Ethics Committee and the Rajanukul Institute Ethics Committee in Thailand prior to the commencement of the study. Participants were fully informed about the study's objectives, procedures, potential risks and benefits, and their right to withdraw at any time without penalty. Written informed consent was obtained from all participants prior to their involvement. To ensure confidentiality and anonymity, each participant was assigned a unique code, and all data were stored securely with access limited to the research team. All interviews were conducted, transcribed, and translated by the primary researcher, who is a native Thai speaker and fluent in English. This approach minimised risks of misinterpretation and preserved cultural and linguistic nuance. Moreover, translations were discussed within the research team to enhance trustworthiness. This addition strengthens transparency around ethical navigation in cross‐language qualitative research.

### 2.4 Data Analysis

Qualitative data from the interview transcripts were analysed using thematic analysis, following the six‐step process outlined by Braun and Clarke (2006). NVIVO software was used to assist in managing and organising the data (Jackson 2019). The six steps were as follows: (1) Familiarisation with the Data: this initial step involved the researcher immersing in the data by repeatedly reading and re‐reading the interview transcripts (both Thai originals and English translations); in this process, all the interviews were transcribed and translated into English by the primary researcher. This process helped gain a deep understanding of the content, context, and nuances of the participants' accounts. (2) Generating Initial Codes: after familiarisation, the researcher began the coding process. This involved systematically identifying meaningful segments of data across the entire dataset relevant to the research questions. These segments were assigned initial codes that captured the essence of the data. At this stage, the focus was on organising the data into smaller, manageable chunks rather than identifying overarching themes. These codes were reviewed by two additional researchers. (3) Searching for Themes: once initial codes were generated, the researcher began to identify potential themes. This involved grouping related codes together and looking for patterns, connections, and broader concepts that developed from the coded data segments. These themes were developed through iterative coding and review that represented overarching ideas that encapsulate key aspects of the participants' experiences related to resilience. (4) Reviewing and Defining Themes: the identified potential themes were then reviewed by all three researchers in relation to the coded extracts and the entire dataset. This step aimed to ensure that the themes were coherent, distinct, and accurately captured the participants' experiences. Some themes were revised, combined, split, or refined throughout this iterative process. (5) Defining and Naming Themes: after the themes were reviewed and validated, they were clearly defined and given concise, descriptive names. Each theme encapsulated a key aspect of the mothers' experiences of resilience. This involved crystallising the meaning and scope of each theme and ensuring that it accurately represented the data it encompassed. (6) Writing the Report: the final step involved compiling the analysis into a coherent narrative. This report presents the story that developed from the data, highlighting the identified themes and their significance. Illustrative quotations from the participants' interviews were selected to provide evidence for the themes and to give voice to the mothers' experiences. The aim was to present a comprehensive and insightful understanding of the data whilst remaining true to participants' perspectives.

Formal member checking of the data analysis was not undertaken. To avoid placing additional burden on these mothers, who already face significant time constraints and caregiving demands. A flexible and iterative team discussion was maintained throughout the analytical process. The researchers engaged in ongoing reflection and discussion to enhance the rigour and trustworthiness of their analysis.

## 3 Results

**FIGURE 1:**
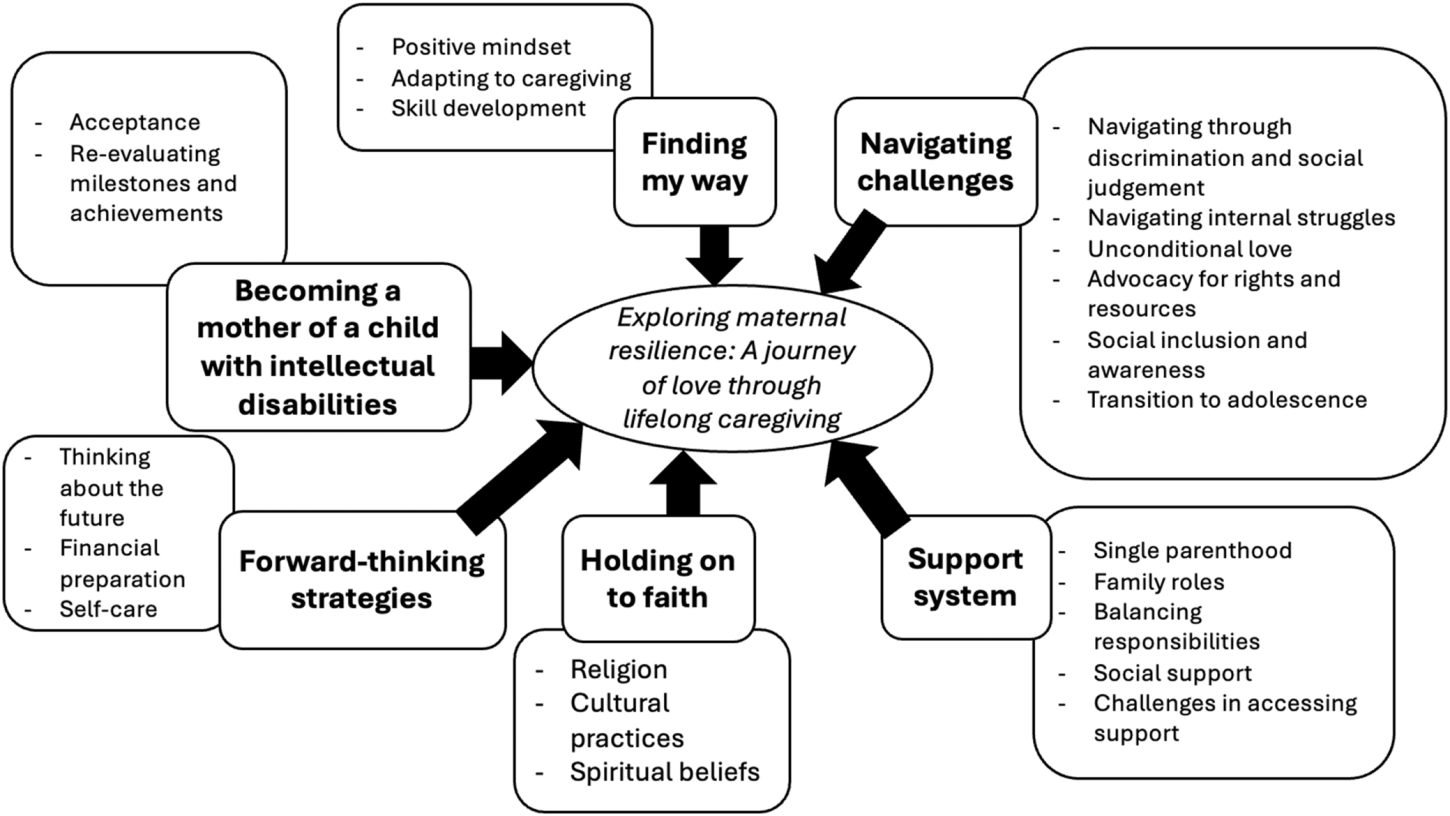
Themes and sub‐themes.

Through the thematic analysis of interview transcripts, six main themes were developed: becoming a mother of a child with intellectual disabilities, finding my way, navigating challenges, support system, holding on to faith, and forward‐thinking strategies. These themes capture the key factors and processes that contribute to resilience amongst the mothers of adolescents with intellectual disabilities. Each theme encompassed a set of sub‐themes, as presented in Figure 1. Table S3 presents a detailed thematic framework based on an analysis of mothers' transcripts. The table is structured into six main themes that developed from the mothers' experiences: “Becoming a mother of a child with intellectual disability,” “Finding my way,” “Navigating through the challenges,” “Support system,” “Holding on to faith,” and “Forward thinking strategies”. Each theme was broken down into more specific sub‐themes and illustrated with direct, poignant quotations from the mothers, providing a rich, qualitative insight into their journeys of acceptance, adaptation, and resilience (see Table S3).

### 3.1 Theme 1: Becoming a Mother of a Child With Intellectual Disability

Mothers described a wide spectrum of emotional experiences during their caregiving journeys, which were shaped by their child's needs and developmental milestones. For many, caregiving brings deep fulfilment alongside considerable challenges, highlighting the complexity of their emotional landscape. This theme encompasses the initial emotional responses to the diagnosis and how love, acceptance, and reframed expectations shaped their early experiences of motherhood.

Acceptance: A crucial aspect of the mothers' emotional journey was achieving acceptance of their child's diagnosis and condition. This process often involves overcoming initial grief, letting go of societal expectations, and embracing their child's unique abilities and inherent worth. For many, acceptance was not a passive resignation but an active process that enabled them to move forward with a more positive and constructive outlook:

“At first, it was difficult to see that my child was not developing like other children, but I refused to feel discouraged. I learned not to be ashamed or compare him to others. My child is healthy and lives with me, which is already a gift. We talk about it positively at home, and that helps us accept and support our child the best way we can.” (Mother 6)

Another mother described her initial stress and subsequent adaptation: “When I first found out that my child was different, I felt extremely stressed and anxious. But in the end, I had to adjust and learn how to take care of her in the best way possible.” (Mother 12).

This adjustment often led to a profound sense of commitment, as Mother 9 stated: “I believe that my strength comes from accepting the reality of the situation and doing my best in my role as a mother.”

Re‐evaluating milestones and achievements: As mothers came to accept their child's condition, they often adjusted their expectations regarding developmental milestones. Instead of adhering to conventional timelines, they learned to celebrate small incremental progress in areas such as communication, self‐care, and social interaction as significant achievements. This re‐evaluation was a source of immense pride and motivation:

“My inspiration comes from seeing my child improve every day. That is what keeps me from giving up… It also brings me happiness to know that I am doing something meaningful for him.” (Mother 7)

The ability to take pride in these unique achievements was crucial for sustaining emotional resilience.

Mother 10 shared: “The most important encouragement comes from my child. Seeing her progress, even in small ways, like walking up the stairs or doing something she couldn't do before, gives me energy and makes me proud.”

Reflecting on past growth also reinforced their dedication, as Mother 12 noted: “What keeps me going is looking back and seeing how far my child has come. Even small steps… show me that our efforts are not wasted.”

### 3.2 Theme 2: Finding My Way

This theme encapsulates mothers' active efforts to navigate the ongoing demands of caregiving. It highlights their resilience through the adoption of adaptive strategies, cultivation of a positive mindset, and development of new skills to support themselves and their children.

Positive mindset: A recurring element in the mothers' narratives was their conscious effort to maintain a positive outlook, even amidst significant adversity. This positive mindset was not merely passive hope but an active coping strategy that imbued their caregiving with purpose and determination. Many mothers emphasised a “never‐give‐up” attitude:

“The most important thing is not giving up. We believe that no matter what situation we face, we must find a way to cope and move forward.” (Mother 9)

This determination was often coupled with a proactive approach, as Mother 10 shared: “A never‐give‐up attitude… I remain committed to caring for my child and making small changes each day to ensure a better future for my child.”

Adapting to caregiving: The mothers consistently discussed the necessity of adapting their caregiving approaches to suit their children's evolving needs, especially as they transitioned through different developmental stages. This adaptation requires continuous learning, flexibility, and willingness to re‐evaluate personal expectations:

“I try to adapt and adjust my caregiving methods to suit my child's age and needs. Although it's not easy, I've learned to look at the positive way and hope that my child will grow up happy.” (Mother 7)

This adaptation often involved shifting focus from conventional milestones to practical life skills.

Mother 12 explained: “I've had to adjust my perspective and lower my expectations. Instead, I focus on self‐sufficiency, such as managing daily routines.”

Skill development: A significant aspect of “finding their way” was that mothers actively fostered independence in their children by teaching them essential life skills. This was described as a gradual, patient, and persistent process tailored to each child's abilities, aimed at preparing them for greater autonomy:

“I involve my child in activities that teach self‐help skills, such as washing clothes, cleaning, or preparing small meals. This helps him practice being independent.” (Mother 7)

However, progress was often slow and required ongoing effort, as Mother 12 acknowledged: “There are still many skills he needs to work on… I keep practicing these skills at a pace that suits him.”

### 3.3 Theme 3: Navigating Through the Challenges

This theme delves into the array of external and internal challenges that mothers encounter. These include societal discrimination, personal emotional struggles, the constant need for advocacy, fostering social inclusion, and managing the complexities of their child's transition into adolescence. Their resilience was evident in how they confronted and managed these complex hardships.

Navigating discrimination and societal judgement: Mothers frequently reported encountering discrimination, stigma, and negative attitudes from society, which evoked feelings of discouragement but often strengthened their resolve to advocate for their children. The experiences of being ostracised or receiving unkind remarks were common amongst them:

“Someone saw my child and moved to another seat. It made me feel discouraged, but at the same time, it gave me the strength to keep fighting… This determination started when people in my own home doubted me… That pressure became a push for me.” (Mother 3)

Persistent negative perceptions were a source of distress. Mother 4 shared: “Society always sees my child negatively… People avoid my child… On difficult days, those harsh glances and judgmental stares make me feel deeply disheartened.”

Navigating internal struggles: Beyond external societal pressures, mothers also contend with significant inner conflicts, including self‐doubt, guilt, sadness, and emotional exhaustion. These struggles were often tied to the immense responsibility for their children's well‐being and the relentless nature of caregiving.

“I do have motivation, but I must admit that sometimes I feel disheartened. These feelings… push me to fight harder… I won't give up.” (Mother 6)

Feelings of emotional exhaustion and isolation were palpable for some. Mother 1 expressed: “I feel extremely stressed. Honestly, I'd love to go to the beach… but I can't. Being with my child 24/7 builds up stress.”

Unconditional love: A powerful and recurring sub‐theme was the unconditional love that mothers felt for their children. This deep emotional bond served as a primary motivator, providing the strength to face ongoing challenges and reaffirming their commitment to caregiving:

“I have him, I love him, and I will take care of him in the best way I can.” This love was intrinsically linked to their perseverance. (Mother 4)

This love often helped mothers overcome their own fears and limitations. Mother 6 shared her transformation: “I wasn't always a strong person… But after having my child, that fear turned into motivation… Every time I faced difficulties; I told myself I had to fight for my child… my love for my child makes me stronger.”

Advocacy for rights and resources: Mothers frequently assume the role of advocates for their children, working to secure the necessary educational, medical, financial, and social support for them. Advocacy is often enacted in everyday public interactions and institutional settings:

“When I take my child to different places, I make sure they receive the rights they deserve, like sitting in designated areas.” (Mother 7)

Advocacy also aimed at broader societal change. Mother 8 noted: “Advocating or voicing my child's needs isn't just about helping my own child. It also helps build understanding in society.”

Social inclusion and awareness: A central concern for mothers was fostering genuine social acceptance and inclusion for their children. They advocated that society view their children as individuals with values beyond mere tolerance:

“Society should foster genuine acceptance and view children with special needs as individuals with value, just like everyone else.” (Mother 10)

Mothers actively worked to raise awareness about the challenges their children faced.

Mother 10 added: “I try to talk about the problems… to raise awareness in society. I hope this leads to equal treatment.”

Transition to adolescence: The entry of their children into adolescence brought intensified emotional demands and new challenges related to managing emotions and behaviour, navigating sexual development, and addressing educational transitions:

“Raising a teenager with intellectual disabilities is challenging. We must be very patient. Sometimes I need to step away, calm myself down.” (Mother 9)

Others faced new behavioural challenges, as Mother 7 described: “My child is growing up and starting to show sexual behaviours… I had to adjust and teach him about sexuality, self‐care, and hygiene.”

Uncertainty about future schooling options was a significant source of distress, especially as children neared the age limits for existing programs.

Mother 2 lamented: “He still can't write… After finishing at Rachanukul, he just stays home because they only accept students until 15 years old.”

### 3.4 Theme 4: Support System

This theme explores the critical role of various support systems in the mothers' lives, including the challenges and strengths associated with single parenthood, the dynamics of family roles, the complexities of balancing multiple responsibilities, the impact of broader social support, and the difficulties encountered in accessing necessary aid.

Single parenthood: Single mothers often express a heightened sense of perseverance and recognise their sole responsibility for caregiving. This requires extraordinary strength and endurance.

“I feel like I have to be more patient than other parents. So much more patient because other parents might have their husbands by their side.” (Mother 1)

Emotional exhaustion was a common experience for single mothers managing multiple children with intellectual disabilities, as Mother 4 shared: “Raising these two children with intellectual disabilities is extremely tiring. It makes me feel quite depressed.”

Despite occasional help from extended family, the ultimate responsibility rested with them.

Family roles: The nature and extent of support within families varied greatly. Some mothers received significant help from new partners who were not the biological fathers of their children:

“My new partner waits until my child comes home, helps with cooking, cleaning… He is the main reason I feel stronger.” (Mother 5)

In some families, caregiving was a collaborative effort. Mother 6 described: “We take turns… The father focuses on earning a living. Together, we raise our child.” However, mothers often bore the primary responsibility for behavioural training.

Balancing responsibilities: Mothers described the intricate act of balancing care for their child with intellectual disabilities with the needs of other family members and household responsibilities. Children with intellectual disabilities often took precedence:

“I prioritise caring for my younger child (with ID) over anyone else because she still needs close supervision.” (Mother 8)

Managing competing demands could be overwhelming. Mother 1 described the chaos: “My youngest child is stubborn, and my other child with intellectual disabilities cries all the time… It makes it impossible to concentrate.”

Social support: Support from outside the immediate family, particularly from peers and healthcare professionals, significantly impacted the mothers' emotional well‐being. Connecting with other parents in similar situations reduced their sense of isolation and offered practical guidance:

“After joining the community here, I met other parents, shared experiences… This community has helped me a lot.” (Mother 4)

Mutual encouragement and understanding were vital. Mother 11 shared: “Meeting other parents… made me feel like I wasn't alone… It felt like making new friends who truly understood me.”

Challenges in Accessing Support: Despite the availability of some support systems, many mothers face difficulties in accessing them. Urban environments sometimes lack strong community networks:

“Since then, we've been in the city, I haven't received any support from the community at all.” (Mother 5)

A perceived decline in traditional community values of mutual support was mentioned. Mother 1 noted: “Society today is not like it used to be. Everyone stays in their own rooms… Society has lost that sense of care.” *Mistrust or cultural dynamics could also hinder engagement with support groups*.

### 3.5 Theme 5: Holding on to Faith

This theme explored how spirituality, religious practices, and cultural traditions were integrated into the mothers' lives, offering comfort, meaning, guidance, and a framework for adapting to their caregiving journeys.

Religion: Religious practices, predominantly Buddhism, were a significant source of strength and solace for many mothers. Daily prayer is a common ritual that provides stability and focus:

“One of the things I do every day is pray and ask for blessings… These practices are not just a source of comfort but also help me reflect and plan my life better… the calmness that comes from praying and making merit gives me the strength.” (Mother 7)

Cultural practices: Cultural traditions were described as resources that supported mothers' emotional regulation and meaning‐making in everyday caregiving. Rather than being rigid obligations, traditions such as temple attendance, merit‐making, and participation in cultural festivals (e.g., Loy Krathong) were selectively maintained and adapted to fit the demands of caring for an adolescent with intellectual disabilities. These practices provided moments of calm, reflection, and hope, and also offered opportunities for learning and social participation for their children.

“It helps my mental health a lot and makes me feel relieved. It calms me down and keeps me peaceful. Once I pray or chant, I feel fine and like everything is going to be okay. It's really more about the mental support it gives me.” (Mother 6)

“These practices give me the space to reflect and plan my life better. I believe the peace I find through prayer and making merit gives me the strength to handle the challenges of parenting and makes me feel like I'm not alone on this journey.” (Mother 7)

“Having a peaceful mind from practicing Dhamma helps me stay patient and handle various situations much better.” (Mother 8)

Mothers often prioritised practical needs over strict adherence to all conventional norms but still found value in upholding traditions, especially during special occasions:

“Lately, we've been going to the temple less often because of our responsibilities… Even so, we still try to find time for special occasions.” (Mother 9)

Spiritual beliefs: Beyond formal religion and cultural rites, personal spiritual beliefs, including belief in guardian spirits or intuition, provided reassurance and a framework for understanding life events:

“Sometimes, if we fail to pay respects to them, my child might fall ill… I truly believe it protects my child well.” (Mother 4)

### 3.6 Theme 6: Forward Thinking Strategies

This theme highlights the proactive and practical strategies mothers employed to foster growth and resilience in themselves and their children, focusing on future planning, financial preparation, and self‐care.

Thinking about the future: Mothers emphasised the critical importance of planning for their children's futures, especially for a time when they might no longer be able to provide care. This involves the creation of opportunities for sustainable living and vocational skills:

“I'm planning to build a future for my child, such as developing jobs they can do on their own. I've started projects like fish farming… sustainable jobs that will suit them in the future.” (Mother 11)

Mother 8 shared a similar hope: “I want them to have opportunities to undergo vocational training so they can develop the ability to support themselves.”

Financial preparation: Financial planning is viewed as a cornerstone of long‐term caregiving. Budgeting, saving, and managing expenses are crucial for securing a child's future and reducing parental stress:

“Preparing for her future is something we focus on. We're trying to save money to cover future expenses… Our main goal is to help her become as independent as possible.” (Mother 12)

Self‐care: Mothers recognised that their well‐being directly impacted their ability to care for their children. Maintaining good physical and mental health was not seen as a luxury but as a responsibility:

“I try to take care of my health and avoid getting sick because if I fall, my child will be directly affected.” (Mother 9)

Long‐term planning also involved making important medical and legal decisions for the child's future quality of life.

Mother 3 worried: “I know I will grow older, and if I ever develop a health issue, it will definitely impact my ability to care for my child… I'm afraid there will be no one to replace me.” This fear added a layer of anxiety to their forward‐thinking strategies.

## 4 Discussion

This study offers a culturally grounded account of how Thai mothers of adolescents with intellectual disabilities sustain their resilience in the face of chronic demands. To our knowledge, this is the first qualitative investigation to specifically focus on this group in Thailand. This shows that resilience is not a static trait but an evolving, everyday practice shaped by appraisal, adaptation, social connection, spirituality, and future planning. Mothers described a movement from initial grief to acceptance and an intentional resetting of developmental expectations. Celebrating small, incremental gains in communication, self‐care, and social participation did not diminish aspirations for their children; rather, it reframed success in ways that were both attainable and motivating. This trajectory is consistent with the broader caregiving literature, in which acceptance and positive reappraisal are linked to better psychological outcomes (Beighton and Wills 2017, 2019; Slattery et al. 2017).

In the UK context, the promotion of resilience often aligns with a neoliberal political agenda aimed at pulling back the Welfare State and fostering independence (Allmark et al. 2014). This is sometimes explicitly tied to initiatives like the UK Government's “big‐society agenda,” which encourages communities to manage their own welfare to avoid the “dependence” associated with state provision (Allmark et al. 2014). Consequently, resilience in policy frequently interpellated the individuals, communities, and private sector as responsible social actors and asks for active citizenship, as opposed to the state organising the social and economic wellbeing (Vilar‐Lluch et al. 2025). This focus on individual and community resilience becomes particularly dangerous when applied to chronic, structural problems like poverty, the cost‐of‐living crisis, and household food insecurity.

However, in the Thai context, the movement from grief to acceptance and reframing expectations appeared to be scaffolded by social and cultural scripts of compassion and letting go, which may make acceptance feel less like resignation and more like an ethical stance toward caregiving (Sethabouppha and Kane 2005; Yiengprugsawan et al. 2012). Resilience was also enacted through agency: mothers spoke about “finding my way” by cultivating a positive mindset, continually tailoring routines, and deliberately teaching daily living skills to foster their children's autonomy. These accounts align with evidence that problem‐focused coping and skill‐building can buffer stress in long‐term care (Jamir Singh et al. 2023). What is notable in our data is the pragmatism with which mothers iterated strategies as children's needs changed through adolescence, suggesting resilience as an ongoing cycle of “appraise to adapt to evaluate” rather than a single turning point (Walsh 2016). This complements international work on parent‐mediated approaches but shows how mothers often generate these techniques informally at home in resource‐variable settings (Duncan et al. 2021; Koly et al. 2021; Taconet et al. 2024).

Simultaneously, narratives made the weight of external and internal stressors visible. Mothers navigated social stigma and subtle exclusion and managed guilt, sadness, and exhaustion that coexisted with love for their child. Unconditional love surfaced not as a sentimental theme but as a stabilising force that helped mothers persist when public spaces felt unwelcoming or when services were difficult to access (Scior 2011; Jansen‐van Vuuren and Aldersey 2020). This limited inclusion and residual traditional beliefs can magnify the social impact of stigma, which helps explain mothers' emphasis on advocacy and awareness (Scior et al. 2020). Their advocacy, whether negotiating with schools, health providers, or officials, was a daily practice aimed at securing rights, resources, and dignity (Rossetti et al. 2021; Smith‐Young et al. 2022). These patterns mirror long‐standing descriptions of “courtesy stigma” affecting families, yet the Thai mothers' emphasis on genuine inclusion (beyond tolerance) points to a shift from mere access to a sense of belonging. The transition to adolescence amplifies these pressures, with new demands regarding behaviour, puberty, sexuality, and service transitions (Ally et al. 2018; Betz 2023; Ramachandran et al. 2025). This echo reports across disability contexts that transition points are particular stress peaks; here, mothers linked transition challenges directly to their own well‐being, underscoring the need for earlier and clearer planning pathways.

Support networks were decisive but unevenly distributed. Single mothers have been found to exhibit heightened perseverance in the face of concentrated responsibility and financial strain (Kim et al. 2023). Within extended families, support varies from indispensable practical help (including by stepparents and grandparents) to conflict that complicates reliance (Ingersoll‐Dayton et al. 2020; Kodyee et al. 2024). Outside the household, peer parents and responsive professionals reduced isolation and provided credible guidance to parents. These accounts are consistent with the well‐documented direct and buffering effects of social support in caregiving (Halstead et al. 2018; Zhong et al. 2020; Tejakum et al. 2022; Chen et al. 2025). However, mothers also narrated barriers in accessing help in urban environments without cohesive neighbourhoods, complex bureaucratic pathways, and variability in service responsiveness. Together, these findings suggest that resilience is partly contingent on structures: where services are fragmented, mothers must expend more effort to maintain a baseline of stability.

Spirituality and culture were integral, rather than peripheral. Many mothers described Buddhist practices (prayer and merit‐making) and personal spiritual beliefs as sources of comfort, acceptance, and emotional regulation. This aligns with the Thai caregiving study of the people with mental health conditions in Thailand, which describes acceptance and compassion framing as a natural part of life (Sethabouppha and Kane 2005).

The role of religion in these mothers' lives is very different from the assumptions made about religion in disability studies in the Global North, which heavily privileges contemporary, seemingly secularised societies (Blackie et al. 2025). Within this paradigm, religion is frequently cast in a negative light and stereotyped as a monolithic force that separates people as “abnormal” and oppresses those with disabilities (Blackie et al. 2025). This bias is built on a secular, modernist assumption that religion is a “backward” superstition that will naturally disappear as people become more economically advanced (Goodley et al. 2012). Consequently, mainstream disability scholars often discount the significance of religious beliefs in shaping the perceptions and experiences of disabled people and their caregivers (Blackie et al. 2025). Applying a “lived religion” perspective which focuses on the everyday practice of belief rather than strict theological doctrine helps to correct this oversight (Blackie et al. 2025).

The identified role of lived religion as a resilience resource (e.g., meaning‐making, emotional regulation, acceptance) may resonate with caregiving in other cultural settings, whilst still recognising the study's findings are grounded in the Thai context. Religion and theology are central ways that many people make sense of the world and their own place within it (Imhoff 2017). Through their faith, individuals are often able to find the strength to manage their pain, utilising a “positive framing” that makes their circumstances feel less distressing and gives them a greater sense of control (Imhoff 2017). In the Indian context, the Hindu doctrine of karma frequently induces an attitude of “tacit acceptance” regarding physical disability (Goodley et al. 2012). Rather than just passive resignation, this spiritual belief can keep a person's faith in a “just world” intact, helping them to understand their own angst and allowing a sense of both “desolation and hope” to be entertained together (Goodley et al. 2012).

Cultural rituals were flexibly adapted to caregiving realities prioritised at key moments and relaxed when daily demands were high, yielding a form of “practical spirituality” that supported endurance without rigid obligation. This resonates with the Thai study in older people highlighting merit‐making and community religious life as resources for wellbeing (Manasatchakun et al. 2016) and extends them to mothers of adolescents with intellectual disabilities. A key cultural coping mechanism is Thum‐jai, the practice of “making peace in your heart” by accepting situations beyond one's control. Far from passive resignation, Thum‐jai is an active spiritual adjustment that fosters emotional stability and protects mental well‐being by relinquishing excessive worry (Mills et al. 2019). This is complemented by spiritual rituals such as prayer, chanting, and meditation, which provide comfort, hope, and practical tools for managing emotional distress (Petcharat and Liehr 2021; Mamom and Daovisan 2022; Klangrit et al. 2025).

Finally, mothers described “forward‐thinking strategies” that made resilience tangible: planning for future living and vocational skills (“when I am gone”), budgeting and saving as a core caregiving task, and attending to their own health as an ethical responsibility for their child. These accounts complement international work, highlighting the importance of early transition planning and financial security for family adaptation (Ally et al. 2018). In our study, future planning functioned as both a practical safeguard and a psychological anchor that reduced uncertainty in the present. In addition to growing confidence, mothers often channel their resilience into proactive advocacy for their children. Furthermore, their engagement in future planning, such as teaching life skills or financial planning, demonstrates adaptive resilience, characterised by preparing for future challenges with intention rather than simply enduring the present (Griffiths et al. 2011). Ultimately, many mothers articulated that the immense challenges of caregiving catalysed profound personal growth, fostering qualities such as patience and a renewed sense of purpose, reinforcing the notion that adversity can be a catalyst for transformation (Minić et al. 2022).

Across themes, the findings converge with wider caregiving literature: acceptance and positive reappraisal as turning mechanisms; the centrality of peer and professional support; the strain of service transitions; and the coexistence of burden with uplifts such as pride and joy (Beighton and Wills 2017). They also extend current knowledge by showing how Thai cultural and spiritual resources channel known resilience processes, how compassion traditions buttress acceptance, how multigenerational households shape both support and conflict, and how temples, peers, and practical wisdom form an ecosystem of care (Sethabouppha and Kane 2005; Gray et al. 2016). Where our results diverge, the differences are instructive. Mothers' reports of persisting public stigma, even alongside policy progress, suggest that attitudinal change lags behind formal provisions, and the emphasis on everyday, relationship‐based advocacy rather than strictly rights‐based contention may reflect local norms that value harmony and incremental negotiation. Taken together, these findings add novel, culturally specific insights into the global caregiving literature and provide a foundation for interventions that respect the particulars of maternal resilience.

The strength of this study lies in its rich and in‐depth exploration of maternal resilience within a distinct cultural context. However, several limitations of this study should be acknowledged. The study involved a small purposive sample of 12 mothers from a single specialised institute in Bangkok. As such, the findings are not generalisable, and the participants' access to support services may distinguish their experiences from those of mothers in less resourced or rural areas. Future studies should address these limitations and expand the scope of this enquiry.

These findings have significant implications for practice, policy, and education in Thailand. Healthcare professionals should develop culturally congruent support that validates mothers' emotional journeys and integrates an understanding of concepts such as thum‐jai. Policymakers must strengthen support systems by improving access to respite care, financial assistance, and vocational training for adolescents with intellectual disabilities and launching campaigns to reduce societal stigma and promote inclusion.

Future research should examine the evolution of resilience over time using longitudinal designs. Including the perspectives of fathers, other caregivers, and adolescents with intellectual disabilities is crucial for gaining a holistic understanding of family resilience. Exploring the experiences of families living in rural or under‐resourced areas of Thailand is particularly important because services and cultural practices may differ significantly.

## 5 Conclusion

This qualitative study provided a nuanced understanding of resilience as a complex, evolving, and culturally grounded process for Thai mothers raising adolescents with intellectual disabilities. The findings illuminate that resilience is not a static attribute but an evolving process that begins with profound emotional adjustment and is sustained through a combination of diverse coping strategies, vital support systems, and proactive advocacy.

Central to their experience is the role of Thai culture and Buddhist faith, which offers a framework for meaning‐making and fosters unique coping mechanisms such as thum‐jai. The mothers in this study demonstrated remarkable strength and adaptability, transforming hardship into personal growth and a deep commitment to their children's futures. By giving voice to their lived experiences, this research underscores the necessity of moving beyond a focus on burden toward a strengths‐based approach. Acknowledging the agency and unique resilience pathways of these mothers is vital for developing compassionate and culturally congruent support systems that empower both mothers and their children to thrive.

## Author Contributions

Dr. Wattana Tejakum conceptualised and designed the study, conducted data collection in Thailand, analysed and interpreted the data, and wrote the first draft of the manuscript. Dr. Maria Truesdale contributed to the conceptualisation of the study, data analysis, and reviewed the manuscript. Prof. Deborah Cairns contributed to the conceptualisation of the study, analysed and interpreted the data, and reviewed the manuscript.

## Funding

This research was funded by the Royal Thai Government Scholarship, under grant PH_G5620.

## Ethics Statement

Data were collected after obtaining ethical clearance from the University of Glasgow Ethics Committee (ID: 200230028) and the Rajanukul Institute Ethics Committee (RI.IRB 011/2566). Written informed consent was obtained from all the participants.

## Conflicts of Interest

The authors declare no conflicts of interest.

## Supporting information


**Table S1:** Demographic information of mothers and their adolescents with intellectual disabilities.

**Table S2:** The interview guide.

**Table S3:** Thematic framework coding.

## Data Availability

The data that support the findings of this study are available from the corresponding author upon reasonable request.
