# Supplementary material for: Exploring Resilience in Mothers of Adolescents With Intellectual Disabilities in Thailand: A Qualitative Study
Source: J Appl Res Intellect Disabil. 2026 Jun 23;39(4):e70271. doi: 10.1111/jar.70271 (PMC13288015; doi:10.1111/jar.70271)
Supplement: Supplementary file 1 — Table S1: Demographic information of mothers and their adolescents with intellectual disabilities. [file JAR-39-e70271-s001.docx]

Table 1: Demographic Information of Mothers and their Adolescents with Intellectual Disabilities

| Mother pseudonym | Age of mother in years | Marital status | Age of an adolescent child with intellectual disabilities in years | Gender of a child with intellectual disabilities | Severity of intellectual disabilities | Number of other dependants (children) | Number of  other  dependants (grandparents) |
| --- | --- | --- | --- | --- | --- | --- | --- |
| 1 | 47 | Single | 18 | Male | Profound | 1 | - |
| 2 | 51 | Single | 12 | Male | Profound | - | - |
| 3 | 47 | Married | 12 | Female | Moderate | 1 | 3 |
| 4 | 40 | Separated | 13, 9 | Both Male | Moderate |  | 1 |
| 5 | 53 | Married | 13 | Female | Mild | - | - |
| 6 | 49 | Married | 14 | Male | Moderate | - | - |
| 7 | 43 | Divorced | 10 | Male | Moderate | 1 | 1 |
| 8 | 54 | Divorced | 18 | Female | Moderate | 2 | - |
| 9 | 32 | Married | 12 | Female | Severe | 1 | - |
| 10 | 54 | Widowed | 18 | Female | Severe | 1 | 2 |
| 11 | 47 | Married | 13 | Male | Mild | 1 | - |
| 12 | 31 | Married | 15 | Male | Moderate | 1 | - |
