# Supplementary material for: Exploring Resilience in Mothers of Adolescents With Intellectual Disabilities in Thailand: A Qualitative Study
Source: J Appl Res Intellect Disabil. 2026 Jun 23;39(4):e70271. doi: 10.1111/jar.70271 (PMC13288015; doi:10.1111/jar.70271)
Supplement: Supplementary file 2 — Table S2: The interview guide. [file JAR-39-e70271-s002.docx]

Table 2 The interview guide

| Preamble: Thanks for joining this study. You decide how much you want to share. If you ever feel uncomfortable, you can let me know and we can stop. Are you ready to continue? | |
| --- | --- |
| 1 | “Can you share your experiences of raising an adolescent with intellectual disabilities?” |
| 2 | “What are some challenges you've faced while parenting your adolescent with intellectual disabilities as they transitioned into adolescence, and how have you managed them?”  (Prompts: Transition child to adult in terms of services, education) |
| 3 | “Can you share any moments when you've felt particularly resilient in your role as a mother of an adolescent with intellectual disabilities?”  (Prompts: What kept you motivated?) |
| 4 | “What kinds of support from your family, community or social circle have helped you in times of difficulty?” |
| 5 | “How do you find a balance between the needs of your adolescent with intellectual disabilities and the needs of the rest of your family?”  (Prompts: Do you have any examples you would like to share?) |
| 6 | “Do you have any cultural practices or traditions that you've found helpful in coping with the unique challenges you face?”  (Prompts: Attending the church or temple or praying) |
| 7 | “How do you think societal attitudes toward intellectual disabilities have impacted your resilience?” |
| 8 | “What personal qualities or strengths do you draw upon when facing the challenges of raising an adolescent with intellectual disabilities?”  (Prompt: Never give up, positive attitude) |
| 9 | “Have you ever felt empowered to advocate for adolescent's needs?” |
| 10 | “Looking back on your experiences, what advice would you give to other mothers in a similar situation within your cultural community?”  (Prompts: What lessons have you learned that you wish you knew when you first started your parenting journey?) |
| 11 | “Are there any comments that you would like to add?” |
