# Supplementary material for: Exploring Resilience in Mothers of Adolescents With Intellectual Disabilities in Thailand: A Qualitative Study
Source: J Appl Res Intellect Disabil. 2026 Jun 23;39(4):e70271. doi: 10.1111/jar.70271 (PMC13288015; doi:10.1111/jar.70271)
Supplement: Supplementary file 3 — Table S3: Thematic framework coding. [file JAR-39-e70271-s003.docx]

**Table 3: Thematic Framework Coding**

| **Main themes** | **Sub-themes** | **Quotations** |
| --- | --- | --- |
| Theme 1: Becoming a mother of a child with intellectual disabilities | Acceptance | Mother 6 shared: *"At first, it was difficult to see that my child was not developing like other children, but I refused to feel discouraged. I learned not to be ashamed or compare him to others. My child is healthy and living with me, that is already a gift. We talk about it positively at home, and that helps us accept and support our child the best way we can."*  Another mother (Mother 12) described her initial stress and subsequent adaptation: *"When I first found out that my child was different, I felt extremely stressed and anxious. But in the end, I had to adjust and learn how to take care of her in the best way possible."*  This adjustment often led to a profound sense of commitment, as Mother 9 stated: *"I believe that my strength comes from accepting the reality of the situation and doing my best in my role as a mother."*  Some mothers admitted to early struggles with negative emotions and self-blame.  Mother 9 recounted: *"At first, I had many negative feelings. When I found out my child was different, I kept asking myself, ‘Did I do something wrong?’ or ‘Why did this happen?’ But as time passed, I started to see that maybe my child was sent to teach me patience and to make me stronger."*  This shift in perspective, focusing on their child’s unique capabilities rather than societal expectations, was pivotal for fostering resilience.  However, acceptance was not universally or immediately achieved.  Mother 2 expressed lingering doubts and a sense of unfairness: *"I still don’t understand why my child turned out this way... Why did this happen to my child? I’ve never even touched alcohol, yet my child has this condition. Meanwhile, others who drink and live recklessly have perfectly healthy kids. It’s unfair."* This highlights that the path to acceptance can be fraught with unresolved emotional conflict for some.  For others, acceptance was cultivated through patience and a focus on daily victories.  Mother 7 explained: *“My patience started from accepting that my child is the way he is. My role is to provide love, understanding, and support him in his own way. I see every problem as something to be solved step by step. Every day that we get through is a small victory... If I have a positive mindset, I can pass that positivity on to my child and others.”* This reframing of success and progress set the stage for re-evaluating traditional milestones. |
|  | Re-evaluating milestones and achievements | Mother 7 articulated this well: *"My inspiration comes from seeing my child improve every day. That is what keeps me from giving up... It also brings me happiness to know that I am doing something meaningful for him."*  Similarly, Mother 11 found that witnessing her child’s independent actions, no matter how small, fuelled her motivation: *"When my child started to do things independently, like walking or speaking, it made me feel incredibly proud and gave me more motivation. It showed me that all my efforts were not in vain."*  The ability to take pride in these unique achievements was crucial for sustaining emotional resilience.  Mother 10 shared: *"The most important encouragement comes from my child. Seeing her progress, even in small ways, like walking up the stairs or doing something she couldn’t do before, gives me energy and makes me proud."*  Reflecting on past growth also reinforced their dedication, as Mother 12 noted: *"What keeps me going is looking back and seeing how far my child has come. Even small steps... show me that our efforts are not wasted."*  This re-evaluation extended to finding joy in seemingly minor improvements, as Mother 12 further explained: *"Every time I see small improvements, like my child picking up objects on their own or being able to do a simple task. It gives me strength and reminds me that our efforts are worthwhile... It’s not always easy but seeing them grow in their own way makes me proud of them and myself."*  While most mothers found strength in this reframing, one mother (Mother 2) expressed regret for not intervening earlier: *"I wish I could turn back time. I should have trained my child more when he was young... It’s my biggest regret that I didn’t do enough back then."*  This highlights the internal pressure and self-critique some mothers experience.  Nevertheless, for the majority, hope for their child’s development and pride in their accomplishments acted as a protective buffer.  Mother 8 stated: *"What keeps me motivated to care for my child is love and hope. I want my child to develop better."* This hope extended to future opportunities, such as employment, as Mother 3 expressed: *"I still have hope that my child will be able to pass the evaluation for the employment project... We have to try."*  The journey of becoming a mother to a child with intellectual disabilities, marked by initial emotional struggles, evolved into a path of acceptance and a redefined understanding of achievement. This transition laid the groundwork for active adaptation and the development of coping strategies, explored in the next theme. |
| Theme 2: Finding my way | Positive mindset | Mother 9 stated: *"The most important thing is not giving up. We believe that no matter what situation we face, we must find a way to cope and move forward."*  This determination was often coupled with a proactive approach, as Mother 10 shared: *"A never-give-up attitude... I remain committed to caring for my child and making small changes each day to ensure a better future for my child."*  Maintaining a positive emotional environment at home was seen as crucial, as mothers believed their emotional state directly impacted their children.  Mother 9 explained: *"A positive attitude is crucial. Even on exhausting or stressful days, we try to smile and laugh because we know that our feelings directly impact our children... The key is to find small joys every day."* Forgiveness and letting go of negativity were also important, as Mother 11 articulated: *"If I dwell on negative thoughts... I will feel miserable... Forgiving and letting go makes me feel lighter and allows me to focus fully on raising my child."*  Some mothers viewed challenges as learning opportunities. Mother 12 advised: *"Don’t be afraid of failure. Every time we face a problem; it’s an opportunity to learn and grow."* For others, inner strength was forged through past hardships.  Mother 2 reflected: *"As a child, I had to fight for everything... Neighbours looked down on us for being poor, but I worked hard to prove them wrong."*  These early experiences cultivated a perseverance that became invaluable in her caregiving role. Resilience was often described as an ongoing process, as Mother 7 noted: *"The times I feel strongest are when I overcome challenges and realise I am making my child’s life better."* |
|  | Adapting to caregiving | Mother 7 shared: *"I try to adapt and adjust my caregiving methods to suit my child’s age and needs. Although it’s not easy, I’ve learned to look at the positive way and hope that my child will grow up happy."*  This adaptation often involved shifting focus from conventional milestones to practical life skills.  Mother 12 explained: *"I’ve had to adjust my perspective and lower my expectations. Instead, I focus on self-sufficiency, such as managing daily routines."*  As children grew, caregiving complexities increased, demanding new strategies for behavioural changes, educational needs, and social integration.  Mother 10 found this particularly challenging: *"The biggest challenge is adapting to both my child’s and my own changes at every stage of life. As she grows, the issues become more complex."*  Physical demands also escalated with the child's growth, as Mother 2 described: *"He’s very strong. Now he’s taller than me, stronger than me. Even his father struggles to manage him sometimes."*  Mental and emotional preparedness for daily uncertainty was key. Mother 9 stated: *"My strength in facing these challenges comes from preparing myself every day. I must have a steady mindset."*  Experimentation with parenting strategies was common, as Mother 2 recounted: *"I have tried everything... I kept trying and changing my approach until I found what worked best."*  Adaptation also entailed re-evaluating personal and professional priorities, with some mothers making significant lifestyle changes.  Mother 12 shared: *"Now, I try to spend more time with my child... I have shifted my role to focus on caregiving."* This journey often led to personal growth and enhanced mental resilience. Mother 5 reflected on her inner strength: *"I think I have a certain level of mental strength that allows me to face the challenges."*  Self-reflection was another vital adaptive tool.  Mother 11 said: *"I often ask myself, ‘Did I do something wrong?’ If I’m confident I didn’t, there’s no need to dwell on it. But if there’s something I should improve, I adjust myself to be better."*  Continuous learning from professionals and independent research was also valued.  Mother 7 emphasized being open to new methods: *"We can’t be like a cup that’s already full. We need to be open to learning new things."*  Mother 12 added: *"Consistent learning is essential because it helps me handle challenges better."*  Building a strong bond with their child, even with communication barriers, was actively pursued, as Mother 6 noted: *"Even if they don’t respond the way we expect, communication builds our connection."* |
|  | Skill development | Mother 7 described her approach: *"I involve my child in activities that teach self-help skills, such as washing clothes, cleaning, or preparing small meals. This helps him practice being independent."*  However, progress was often slow and required ongoing effort, as Mother 12 acknowledged: *"There are still many skills he needs to work on... I keep practicing these skills at a pace that suits him."*  Early intervention was highlighted by some. Mother 7 recounted: *"I started taking my child to therapy and special education programs from a young age... But as she grows, new challenges arise."*  Consistency and repetition in training were deemed crucial. Mother 10 emphasized: *"I believe that consistency in training is the most important part... we need to practice at home too."*  Mothers adopted a step-by-step approach, focusing on practical skills like personal hygiene and household chores, aiming to reduce long-term reliance on others.  Mother 4 shared: *"I focus on the present and help my child improve little by little each day. I want them to learn how to take care of themselves... But sometimes, I push too hard... I have to remind myself that some things take time."*  This focus on skill development was a proactive strategy to prepare children for the future, even as mothers navigated ongoing challenges. |
| Theme 3: Navigating through the challenges | Navigating through discrimination and societal judgement | Mother 3 recounted an experience on a bus: *"Someone saw my child and moved to another seat. It made me feel discouraged, but at the same time, it gave me the strength to keep fighting... This determination started when people in my own home doubted me... That pressure became a push for me."*  Persistent negative perceptions were a source of distress. Mother 4 shared: *"Society always sees my child negatively... People avoid my child... On difficult days, those harsh glances and judgmental stares make me feel deeply disheartened."*  Mother 6 added: *"Some neighbours assume that all children with intellectual disabilities are the same... It’s heartbreaking that people still think this way."*  Mother 11 tried to remain resilient: *"The hardest thing is dealing with how others look at and talk about my child... But I try not to let it affect me."*  These experiences often led to anger and frustration, particularly when witnessing direct rejection.  Mother 3 described her anger: *"I remember sitting on the bus, and when someone saw my child, they immediately moved to another seat. I felt frustrated and angry."*  Mother 1 shared a painful memory: *"A seven-year-old once called my child a ‘ghost child’... I wanted to yell at them, but I held back... I was already overwhelmed with anger and sadness."*  Such societal judgments deeply affected the mothers' emotional well-being and often intertwined with their internal struggles. |
|  | Navigating internal struggles | Mother 6 captured the coexistence of motivation and disheartenment: *"I do have motivation, but I must admit that sometimes I feel disheartened. These feelings... push me to fight harder... I won’t give up."*  Feelings of emotional exhaustion and isolation were palpable for some. Mother 1 expressed: *“I feel extremely stressed. Honestly, I’d love to go to the beach... but I can’t. Being with my child 24/7 builds up stress.”*  The demanding nature of caregiving was acknowledged by Mother 5: *"Even though I love my child deeply, I still have moments of frustration. Sometimes, I feel annoyed. Sometimes, I feel tired. It’s natural... There are moments when I feel like I’ve had enough, but I keep going."*  Mothers developed personal coping strategies to manage these internal battles. Self-encouragement was key for Mother 7: *"Do not give up when facing challenges, because every problem has a solution."*  Emotional detachment from external criticism helped others. Mother 2 stated: *"When my child has a meltdown in public, I tell myself not to look at other people. I focus only on my child. I don't care about what others think."*  Mother 11 echoed this: *"I try not to let other people’s opinions affect me."*  Maintaining a positive mindset was a conscious choice. Mother 11 elaborated: *"Looking at the world positively is important... I choose to forgive and not take others’ comments to heart."*  This internal fortitude was often sustained by a profound connection with their child. |
|  | Unconditional love | Mother 4 simply stated: *"I have him, I love him, and I will take care of him in the best way I can."* This love was intrinsically linked to their perseverance.  Mother 7 explained: *"The love and understanding I have for my child are what drive me to keep fighting... The encouragement I create for myself, the love I have for my child, is the driving force that keeps me going."*  This love often helped mothers overcome their own fears and limitations.  Mother 6 shared her transformation: *"I wasn’t always a strong person... But after having my child, that fear turned into motivation... Every time I faced difficulties; I told myself I had to fight for my child... my love for my child makes me stronger."*  This profound love often translated into active advocacy. |
|  | Advocacy for rights and resources | Mother 7 emphasized protecting her child’s rights in daily life: *"When I take my child to different places, I make sure they receive the rights they deserve, like sitting in designated areas."*  Advocacy also aimed at broader societal change. Mother 8 noted: *"Advocating or voicing my child’s needs isn’t just about helping my own child. It also helps build understanding in society."*  Accessing financial aid and government support was a common challenge requiring active pursuit.  Mother 2 recounted: *"I received financial aid... I also advised the father to apply for disability rights for him, so we could access free medical care."*  While resources like disability cards were helpful, navigating the system was often arduous, as Mother 6 acknowledged: *"These benefits make a significant difference."*  Advocacy was also personally empowering.  Mother 7 stated: *"I always ask myself, ‘If I don’t speak up for my child, who will?’ Sometimes, explaining to others... makes me feel stronger and more determined."*  Mothers also advocated for systemic improvements, such as better daycare options, as Mother 8 suggested: *"Special-needs daycare centres in Bangkok should operate like regular schools."* |
|  | Social inclusion and awareness | Mother 10 expressed: *"Society should foster genuine acceptance and view children with special needs as individuals with value, just like everyone else."*  Mothers actively worked to raise awareness about the challenges their children faced.  Mother 10 added: *"I try to talk about the problems... to raise awareness in society. I hope this leads to equal treatment."*  They often had to intervene directly to ensure inclusion, for instance, in schools.  Mother 4 recounted challenging a teacher: *"There was a time in kindergarten when my child was not allowed to participate... The teacher said it was because my child was special needs... I had to ask, ‘Why not let my child join?’"*  Participation in structured activities like the Special Olympics was seen as vital.  Mother 11 shared: *"My child participated in the Special Olympics... It was such a valuable experience. My child was incredibly proud... These activities don’t just help with personal development; they allow children to make friends."*  Mothers made efforts to create an appearance of normalcy to prevent discrimination, while also finding encouragement in moments of societal empathy. Mother 8 said: *"When some people in the community understand and don’t discriminate against my child... These moments give me a lot of encouragement."*  Societal attitudes had a dual effect, as Mother 12 noted: *"The negative side is that it can be exhausting... on the positive side, it pushes me to be more patient and prepared."*  Acts of kindness were particularly heartening for Mother 8: *"When I see some people in society naturally understanding and offering support... it gives me a lot of encouragement."* |
|  | Transition to adolescence | **i) Managing emotions and behaviour:** Patience and emotional self-regulation were key strategies.  Mother 9 shared: *"Raising a teenager with intellectual disabilities is challenging. We must be very patient. Sometimes I need to step away, calm myself down."*  Mother 11 added: *"Staying calm is the most important thing... If we remain calm, they will learn to regulate their emotions too."*  Some found motivation by comparing their situations with others facing greater hardships, as Mother 3 did: *"I see other parents whose children are bedridden... When I see them fighting... I think, why can’t I?"*  Controlling one's own emotional reactions was crucial.  Mother 9 explained: *"If I explode with frustration, it only makes things worse."* Positive discipline was preferred over punishment.  Mother 9 advocated: *"Discipline should be a teaching tool... Using physical discipline isn’t effective."*  **ii) Navigating sexual development:** This brought new anxieties.  Mother 5 worried about safety and exploitation: *"I worry that someone might take advantage of her. If she gets pregnant, it will be a huge problem."*  Others faced new behavioural challenges, as Mother 7 described: *"My child is growing up and starting to show sexual behaviours... I had to adjust and teach him about sexuality, self-care, and hygiene."*  **iii) Next steps in education:** Uncertainty about future schooling options was a significant source of distress, especially as children neared the age limits for existing programs.  Mother 2 lamented: *"He still can’t write... After finishing at Rachanukul, he just stays home because they only accept students until 15 years old."*  Mother 3 worried: *"Next year, when he turns 15, he will have to leave Rachanukul... I don’t know where to send him next."* |
| Theme 4: Support system | Single parenthood | Mother 1 conveyed this: *"I feel like I have to be more patient than other parents. So much more patient because other parents might have their husbands by their side."*  She also described moments of profound helplessness, especially during medical emergencies: *"I felt most helpless when my child was sick... I had no one to help carry him to the hospital... during a seizure, I was alone."*  Emotional exhaustion was a common experience for single mothers managing multiple children with *intellectual disabilities*, as Mother 4 shared: *"Raising these two children with intellectual disabilities is extremely tiring. It makes me feel quite depressed."*  Despite occasional help from extended family, the ultimate responsibility rested with them.  Mother 7 stated: *"Taking care of my child is something I have to do entirely on my own... in the end, I am the only one who provides full-time care."*  Past hardships often forged resilience, as Mother 8 reflected: *"Being a mother has made me stronger... I see these obstacles as experiences that have made me tougher."*  The sense of duty was paramount: *"The thought that keeps me going every day is: ‘If I don’t love and take care of my child, who will?’"* (Mother 8). |
|  | Family roles | Mother 5 shared: *"My new partner waits until my child comes home, helps with cooking, cleaning... He is the main reason I feel stronger."*  Mother 12 added: *"My current partner has helped raise my child... He treats my child as his own... Not having to care for my child alone has reduced my stress."*  In some families, caregiving was a collaborative effort. Mother 6 described: *"We take turns... The father focuses on earning a living. Together, we raise our child.”*  However, mothers often bore the primary responsibility for behavioural training.  Mother 2 noted: *"At home, my husband doesn’t really train our child... I’m the one who bathes him and teaches him."* Support from extended family, like grandparents, could be pivotal, even if understanding developed over time.  Mother 4 explained: *"At first, my mother didn’t understand... But when I explained... she began to support us... She now encourages us."* Family unity, love, and mutual support were key.  Mother 9 emphasized: *"Love, understanding, and helping each other are the heart of keeping our family moving forward."* A supportive partner was often an essential source of emotional stability.  Mother 9 continued: *"My partner is my greatest source of encouragement. If he sees me exhausted... he steps in."*  However, some faced judgment from relatives, adding to their stress.  Mother 3 recounted: *"I was criticised a lot... When I stayed at home, everyone was stressed."* |
|  | Balancing responsibilities | Mother 8 stated: *"I prioritise caring for my younger child (with intellectual disabilities) over anyone else because she still needs close supervision."*  Mother 6 added: *"My child with special needs requires extra attention. My husband... can take care of himself."*  Managing competing demands could be overwhelming.  Mother 1 described the chaos: *"My youngest child is stubborn, and my other child with intellectual disabilities cries all the time... It makes it impossible to concentrate."*  Strategies for balance included making compromises for inclusive family experiences.  Mother 10 explained: *"I try to treat all my children equally... I’ll buy food to bring home so everyone can participate."*  Structuring time effectively was also important.  Mother 12 noted: *"I organise the schedule clearly to avoid feelings of neglect."*  Some mothers made efforts to maintain their marital relationships.  Mother 12 shared: *"Sometimes we find opportunities to spend time together... This helps us maintain our personal relationship."* |
|  | Social support | Mother 4 reflected: *"After joining the community here, I met other parents, shared experiences... This community has helped me a lot."*  Mutual encouragement and understanding were vital.  Mother 11 shared: *"Meeting other parents... made me feel like I wasn’t alone... It felt like making new friends who truly understood me."*  Recognizing the value of asking for help was a shift for some.  Mother 7 realized: *"If I couldn't take care of my child, who would?*  *This made me realise that relying on others is sometimes unavoidable."*  Mother 8 encouraged others: *"If I need help, I should not hesitate to ask... You are not alone."*  Healthcare professionals were essential allies.  Mother 12 stated: *"Regular medical visits are essential... Consulting doctors for treatment... can help alleviate these issues."*  Financial support, where available, eased burdens.  Mother 1 received an allowance from her ex-husband: *"This financial aid allows me to care for my child full-time, and that is my happiness."*  Mother 6 managed through careful financial planning: *"We live simply... We use what we have wisely."*  Special education centres like Rachanukul provided crucial practical and emotional support.  Mother 3 found: *"The community at Rachanukul is much better than my home environment. Here, we share, discuss, and solve problems together."* |
|  | Challenges in Accessing Support | Mother 5 noted: *"Since then, we've been in the city, I haven't received any support from the community at all."*  A perceived decline in traditional community values of mutual support was mentioned.  Mother 1 noted: *"Society today is not like it used to be. Everyone stays in their own rooms... Society has lost that sense of care."* Mistrust or cultural dynamics could also hinder engagement with support groups.  Mother 3 expressed discomfort: *"Even in parent support groups... I don’t feel comfortable sharing personal issues because I don’t trust that they won’t gossip about me."* Similarly,  Mother 6 felt a lack of neighbourhood interaction: *"In my neighbourhood, no one really comes to talk or offer help."*  Economic hardships also posted significant challenges, with some mothers leaving jobs to provide care, leading to financial strain and loss of social connections, as Mother 9 experienced.  Mother 4 expressed the daily struggle: *"Having money would make things much easier... just enough to eat, pay rent, and survive."* |
| Theme 5: Holding on to faith | Religion | Mother 7 explained: *"One of the things I do every day is pray and ask for blessings... These practices are not just a source of comfort but also help me reflect and plan my life better... the calmness that comes from praying and making merit gives me the strength."*  Visiting temples and making merit (performing good deeds) helped find peace.  Mother 3 shared: *"I go to the temple to make merit... From what I’ve done so far, it has helped. It makes me feel at peace."*  Religious activities like listening to Dharma teachings and meditating cultivated inner peace and patience.  Mother 12 noted: *"These religious practices... give me time to reflect on myself and regain emotional strength... They help me find hope and patience."*  Mother 11 found strength in Dharma: *"I listen to Dharma teachings and meditate every day. It helps me stay calm."* Religion provided a sense of control and emotional regulation.  Mothers prayed for their children’s well-being, as Mother 9 did: *"When I’m there, I always pray for my child’s good health and development."*  Sacred objects like Buddha statues also served as reminders of faith, as Mother 7 described: *"I have a Buddha statue and an image of the Emerald Buddha at home… and to improve in their communication skills."*  Some mothers observed positive effects on their children in religious settings.  Mother 2 noted: *"When I take them into the temple, they don’t act out. They seem calmer."* |
|  | Cultural practices | Cultural practices were described as resources that supported mothers’ emotional regulation and meaning-making in everyday caregiving.  Mother 6 mentioned: *"It helps my mental health a lot and makes me feel relieved. It calms me down and keeps me peaceful. Once I pray or chant, I feel fine and like everything is going to be okay. It’s really more about the mental support it gives me."*  Mother 7 mentioned: *"These practices give me the space to reflect and plan my life better. I believe the peace I find through prayer and making merit gives me the strength to handle the challenges of parenting and makes me feel like I’m not alone on this journey."*  Mother 8 mentioned: *"Having a peaceful mind from practicing Dhamma helps me stay patient and handle various situations much better."*  Mother 9 mentioned: *"Lately, we’ve been going to the temple less often because of our responsibilities... Even so, we still try to find time for special occasions."*  For some, cultural practices were supportive but not foundational, with love and daily effort being paramount.  Mother 9 stated: *"These cultural practices help to some extent... but they are not something I strictly follow... I believe more in love, understanding, and the daily effort."*  Others adapted cultural practices to promote their children’s learning and well-being.  Mother 12 described her child’s engagement in the Loy Krathong festival: *"They learned the process... These activities not only brought happiness but also helped my child develop a sense of learning and calmness."* |
|  | Spiritual beliefs | Mother 4 believed in household spirits: *"Sometimes, if we fail to pay respects to them, my child might fall ill... I truly believe it protects my child well."*  Spirituality often centered on positive intentions.  Mother 6 shared: *"I don’t hold onto negative beliefs that make my mind feel heavy. When I pray, I ask for peace, good health for my family."*  A strong sense of intuition was developed by some.  Mother 11 called it her "sense": *"When they’re about to get sick, I sometimes dream about them... having these signs helps me feel like I am doing my best."*  Some mothers initially turned to fortune-telling, like Mother 1, who found predictions came true: *" When I was 21, I went to a fortune teller … He told me that by the time I turned 22, I would have wealth, a house, and someone from far away would come into my life. Three to four months later, I … met my child’s father. He gave me gold, and after six months, he bought me a house just like the fortune teller predicted!"*  However, others, like Mother 8, shifted away from such beliefs over time: *"I used to be interested in magic and fortune-telling, but as time passed, I realized that everything depends more on my own actions than on fate."*  Spiritual practices often led to emotional healing and letting go of negative emotions.  Mother 5 described embracing forgiveness after a betrayal: *"By letting go of anger and embracing forgiveness, I became more cheerful, and my relationships with others improved."* |
| Theme 6: Forward thinking strategies | Thinking about the future | Mother 11 was actively developing projects: *"I’m planning to build a future for my child, such as developing jobs they can do on their own. I’ve started projects like fish farming... sustainable jobs that will suit them in the future."*  Mother 8 shared a similar hope: *"I want them to have opportunities to undergo vocational training so they can develop the ability to support themselves."*  The goal was often independence, as Mother 7 stated: *"My goal is for my child to become as independent as possible... I am confident that he has his own strengths."* |
|  | Financial preparation | Mother 12 explained: *"Preparing for her future is something we focus on. We’re trying to save money to cover future expenses... Our main goal is to help her become as independent as possible."* |
|  | Self-care | Mother 9 emphasized: *"I try to take care of my health and avoid getting sick because if I fall, my child will be directly affected."*  Long-term planning also involved making important medical and legal decisions for the child's future quality of life.  Mother 3 worried: *"I know I will grow older, and if I ever develop a health issue, it will definitely impact my ability to care for my child... I’m afraid there will be no one to replace me."*  This fear added a layer of anxiety to their forward-thinking strategies. |
